# 

**Published:** 1903-05-23

**Authors:** 


### NOTICE TO CORRESPONDENTS.

All MSS., Letters, Books for Review, and other matters intended for the Editor should be addressed The Editor, "The Hospital," The Hospital Buildings, 28 & 29 Southampton Street, Strand, W.C.

The Editor cannot undertake to return rejected MS., even when accompanied by stamped directed envelope.

**Notice to Advertisers and Subscribers.**

All Advertisements, Orders for Copies of The Hospital, and Business Communications should be addressed to **THE MANAGER, (Not to the Editor)**, The Hospital, 28 & 29 Southampton Street, Strand, London, W.C.

The Cost of Subscription, post free, is as follows.—Per week, 3½d.; per quarter, 3s. 9d.; per half-year, 7s. 6d.; per annum, 15s. Foreign and Colonial:—Per annum, 19s. 6d., payable, in all cases, in advance.

**NOTICE.—Vol. XXXIII. of "The Hospital" (October 1902 to March 1903) in handsome cloth binding, will shortly be on sale at the office of this Journal, price 7s. 6d. Cases for binding the Numbers contained in this Volume 1s. 6d. Index to Vol. XXXIII., 2½d., post free.**

**The monthly part for May is now ready. Price 1s., by post 1s. 3d.**

### Scraps and Gleanings.

The Local Government Board have recognised the right of Poor Law medical officers in Ireland to an official holiday.

The number of cases of ophthalmia at the schools of the Central London School District at Hanwell has increased to nearly 180.

Owing to the increase of madness among dogs in Vienna and South Austria, all exhibitions there of dogs and cats are prohibited.

During the last three months nearly 7,400 sparrows were destroyed by a Sparrow Club in Essex as being pests to the farmers and gardeners.

Statistics show that during the past quarter there was a falling off of 10 per cent. from the normal Leeds marriage rate. Depression of trade is the cause assigned.

An anonymous gift of £1,000 has been received by Colonel Vaughan for the Cardiff Infirmary for the purpose of building a pavilion for the treatment of lupus and other diseases.

The Hackney Borough Council have agreed to contribute £10,000 towards the cost of purchasing Springfield Estate, Upper Clapton, providing the London County Council will purchase the ground and maintain it as a public park.

According to the official statement the number of paupers who were in receipt of public relief within the Metropolitan area for the week ending the last day of April was 109,210. For the corresponding week of last year the total was 104,060.

### The Student of Medicine.

### APPOINTMENTS.

**Comyns Berkeley**, M.B., B.C.Cantab., M.R.C.P.Lond., appointed Assistant Obstetric Physician to the Middlesex Hospital. **Samuel Blakely**, M.D.King's Coll.Aberd., M.R.C.S.Eng., appointed House Surgeon to the Royal Victoria Hospital, Belfast. **T. Harrison Butler**, M.A., M.D., B.Ch.Oxon., M.R.C.S., L.R.C.P., appointed Assistant Surgeon at the British Ophthalmic Hospital, Jerusalem. **Philip D. Cogswell**, M.R.C.S.Eng., L.R.C.P.Lond., appointed Certifying Factory Surgeon for the Broughton Astley District, Leicester. **Frederick Robert Cosgrave**, M.D.Dub., B.Ch., appointed Certifying Factory Surgeon for the Burton-in-Kendal District, Westmorland. **John Griffiths**, M.R.C.S.Eng., L.S.A., appointed Certifying Factory Surgeon for the Llandrindod Wells District, Radnor. **Maynard Horne**, M.A., M.B., appointed Honorary Physician to the Margaret Street Hospital for Consumption and Diseases of the Chest (for out-patients). **Walter L. Hubbard**, M.D.Brux., M.R.C.S., L.R.C.P.Lond., appointed Certifying Factory Surgeon for the Edenbridge District, Kent. **G. Cordy Jeaffreson**, M.R.C.S., L.R.C.P.Lond., appointed Certifying Factory Surgeon for the Framlingham District, Suffolk. **George Mowatt**, M.B., Ch.B.Aberd., appointed Police Surgeon of the Borough of Bolton. **Chas. H. Godfrey Ramsbottom**, M.D.Vict., Ch.B., appointed Certifying Factory Surgeon for the Bungay District, Suffolk, and the Ditchingham District, Norfolk. **J. B. O. Richards**, L.R.C.P.&S.Edin., L.F.P.S.Glasg., appointed Certifying Factory Surgeon for the Wadebridge District, Cornwall. **Thomas Edward Richards**, M.B., Ch.B.Edin., appointed Certifying Factory Surgeon for the Abercrave District, Brecon. **James Ritchie**, M.D., C.M., D.P.H.Aberd., appointed Certifying Factory Surgeon for the Old Deer District, Aberdeen. **F. C. Rundle**, L.R.C.P., L.R.C.S.Edin., appointed District Medical Officer of the Tenterden Union. **Mrs. Scarlett-Synge**, M.D.Brux., L.S.A., L.M., appointed Medical Officer to the Government Normal College, Bloemfontein, and to the High School, Bloemfontein. **Sidney Wacher**, F.R.C.S.Eng., L.R.C.P.Lond., appointed Certifying Factory Surgeon for the Canterbury District, Kent. **L. E. Whitaker**, M.R.C.S.Eng., L.R.C.P.Lond., appointed Certifying Factory Surgeon for the Diss District, Norfolk. **Thos. Sanders Worboys**, M.R.C.S., L.R.C.P.Lond., appointed Medical Officer and Public Vaccinator for the Winterton District of the Glanford Brigg Union. **W. S. Wright**, M.R.C.S., L.R.C.P.Lond., appointed Certifying Factory Surgeon for the Wool District, Dorset.

### "The Hospital" Institutional Library.

"Hospitals and Asylums of the World." 4 Vols., with a Portfolio of Plans, complete £12 12s.

"Burdett's Hospitals and Charities, 1902." 5s.

"Burdett's Official Nursing Directory, 1903." 3s. net.

"Cottage Hospitals, General, Fever, and Convalescent." 10s. 6d.

"Uniform System of Accounts for Hospitals and Public Institutions." New Edition. 4s. net.

"Hospital Expenditure: The Commissariat." 2s. 6d.

"A Legal Handbook for the Use of Hospital Authorities." 2s. 6d.

"The Cottage Hospital Case Book or Register of Patients." 10s. and 12s. 6d.

"The Furnishing and Appliances of a Cottage." 6d.

All these are published by the Scientific Press, Ltd., and may be obtained through any bookseller, or direct from the publishers, 28 and 29 Southampton Street, Strand, London, W.C.

### THE MIDWIVES ACT

As this Act requires the registration of all Midwives, and demands that they must possess certain qualifications to be so registered, it becomes imperative that those Nurses who are about to take up this important branch of nursing should obtain the latest and best works on the subject.

### A PRACTICAL HANDBOOK OF MIDWIFERY,

By FRANCIS W. HAULTAIN, M.D.

(Lecturer on Midwifery, Edinburgh School of Medicine), has been thoroughly revised and brought up to date with the object of making it the **best text-book**, and as we feel that this purpose has been achieved we confidently recommend the book to Nurses who are training to become Midwives, as well as to the qualified Midwife, who will find it of the greatest assistance to her in her work.

Bound in leather gilt, price **6/-; 6/3,** post free.

**The following Handbooks will also be found to be exceedingly useful:—**

### THE MIDWIVES' POCKET-BOOK,

By HONNOR MORTEN, contains an **Explanatory Chapter on the Midwives Act,** and also full particulars of the London Obstetrical Society's Examination.

Price **1/-,** post free.

### GYNÆCOLOGICAL NURSING,

By G. A. HAWKINS-AMBLER, F.R.C.S., will prove valuable to Nurses who are called upon to nurse a gynæcological case.

Price **1/-,** post free.

*These books are obtainable of all Booksellers and are published by*

THE SCIENTIFIC PRESS, LTD., 28 and 29 Southampton Street, Strand, London, W.C.,

*who will send their latest Catalogue of Nursing Manuals, post free, to any Nurse on application.*

